# Necrobiosis lipoidica of the breast associated with Crohn’s disease: a case report

**DOI:** 10.1186/s13256-022-03698-9

**Published:** 2023-01-03

**Authors:** Awatef Kelati, François Durupt, Brigitte Balme

**Affiliations:** 1 Dermatology Department, University Hospital Cheikh Khalifa, Mohammed VI University of Health Sciences (UM6SS), Casablanca, Morocco; 2 Dermatology Department, Hospital Croix Rousse, Lyon, France; 3 Anatomo-Pathology Department, University Hospital Lyon Sud, Pierre-Bénite, France; 4 UM6SS-Anfa City-Bld Mohammed Taieb Naciri, Commune Hay Hassani, 82403 Casablanca, Morocco

**Keywords:** Necrobiosis lipoidica, Breast, Crohn disease, Case report

## Abstract

**Background:**

Necrobiosis lipoidica located to the breast; without evidence of glucose intolerance, is extremely rare, and its association to Crohn’s disease is not usual.

**Case presentation:**

We report an interesting case of an association of necrobiosis lipoidica of the breast and Crohn’s disease in a 54-year-old Moroccan woman. Skin necrobiotic changes are a characteristic feature in necrobiosis lipoidica, but they are exceptional in metastatic Crohn’s disease, since there are only three published cases of necrobiotic skin lesions on the lower leg resembling erythema nodosum in metastatic Crohn’s disease.

**Conclusions:**

On the basis of this rare observation, necrobiosis lipoidica without evidence of glucose intolerance should be recognized as a possible cutaneous manifestation or association of Crohn’s disease.

## Introduction

Necrobiosis lipoidica (NL) is a rare granulomatous noninfectious skin disease of unknown etiology and pathogenesis [1]. It was usually described in patients with diabetes mellitus. Rare cases of familial NL without impaired glucose tolerance have been published [2]. NL was also reported over surgical scars (appendectomy scar in a patient having morphea) [3].

Typical, NL lesions occur on the pretibial area as distinctive plaques with yellowish atrophic center and violaceous indurated periphery [4]. Microscopically, NL is typified by palisading necrobiotic non-caseating granulomas aligned parallel to the skin surface. Necrobiosis consists of eosinophilic, swollen, and degenerated collagen, and vascular changes are often seen [4, 5].

Therapeutic options are numerous, including topical or intralesional steroids, topical tacrolimus, photochemotherapy, antimalarial drugs, photodynamic therapy, fumaric acid, thalidomide, pentoxifylline, heparin injections, tumor necrosis factor (TNF) inhibitors, and excision followed by a graft. No treatment option is constantly effective [1].

Moreover, the skin is the most common site of extraintestinal involvement of Crohn’s disease (CD) (22–44% of patients), and skin lesions may be present during, after, and in rare cases, before the active stage of CD. Their exact etiology remains unknown; it is probably a multifactorial response due to immune mechanisms with a granulomatous type IV hypersensitivity reaction [6].

Cutaneous manifestations of CD have variable morphology [6]. The currently accepted classification is based on pathogenic mechanisms [7]: similarly to the pathogenic mechanism of CD, CD-specific manifestations are granulomatous (fissures, fistulae, oral involvement), and granulomatous lesions in a discontiguous site from the gastrointestinal tract are considered as metastatic CD. Reactive cutaneous manifestations arise from a different pathogenic mechanism (erythema nodosum, pyoderma gangrenosum, oral aphthae, erythema multiforme, cutaneous vasculitis, and so on); there are also cutaneous manifestations that are associated with CD, but without a well-described pathogenetic mechanism (palmoplantar pustulosis, vitiligo, palmar erythema, hidradenitis suppurativa) [7].

## Case presentation

We report a case of a 54-year-old Moroccan woman with a 10-year history of CD, for which a small bowel resection was performed 9 years ago, and good disease control was achieved with azathioprine. She presented with a 1-year history of asymptomatic erythematous lesion on the left breast with no history of glucose intolerance, family history of similar lesions, cutaneous disease, or other systemic symptoms.

**Fig. 1:**
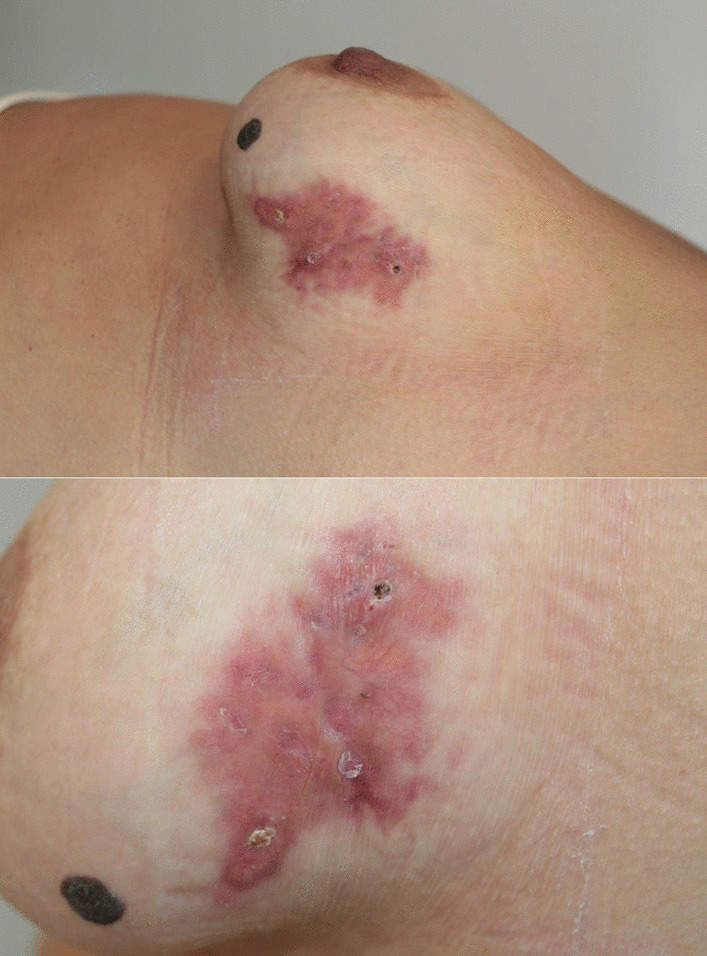
Well-demarcated shiny orange-yellow infiltrated plaque of 6 cm in length on the left breast, with prominent telangiectasias and atrophic areas

Findings from the physical examination revealed a well-demarcated shiny orange-yellow infiltrated plaque of 6 cm in length on the left breast, with prominent telangiectasias and atrophic areas. No other cutaneous lesions or lymphadenopathies were observed (Fig. 1).

**Fig. 2:**
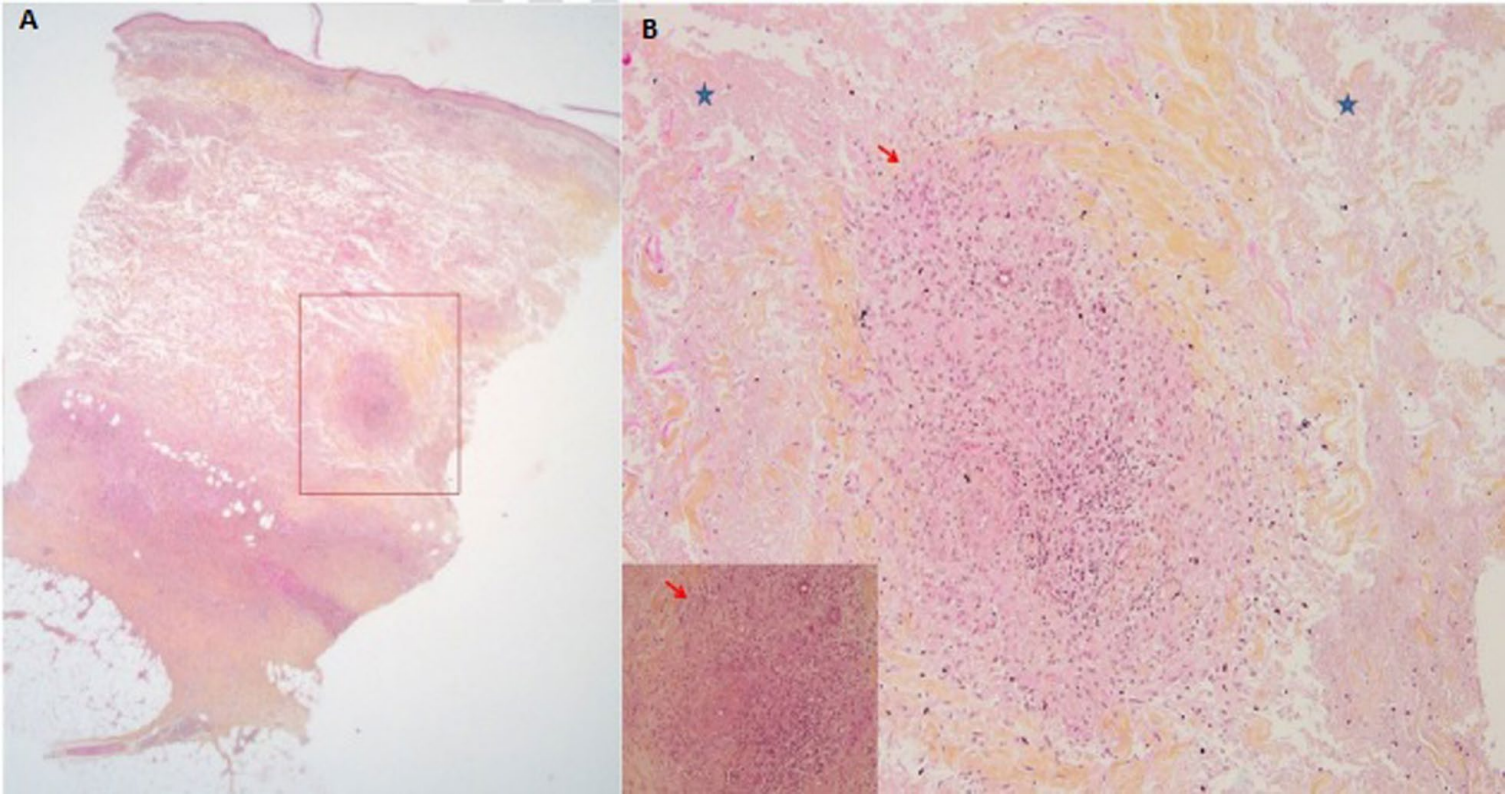
(Hematoxylin and eosin safran stain; original magnification: **A **×4. **B** ×20): Palisading non-caseating interstitial granulomatous dermatitis (red rectangle  and  arrows) with diffuse necrobiosis (blue star)

**Fig. 3:**
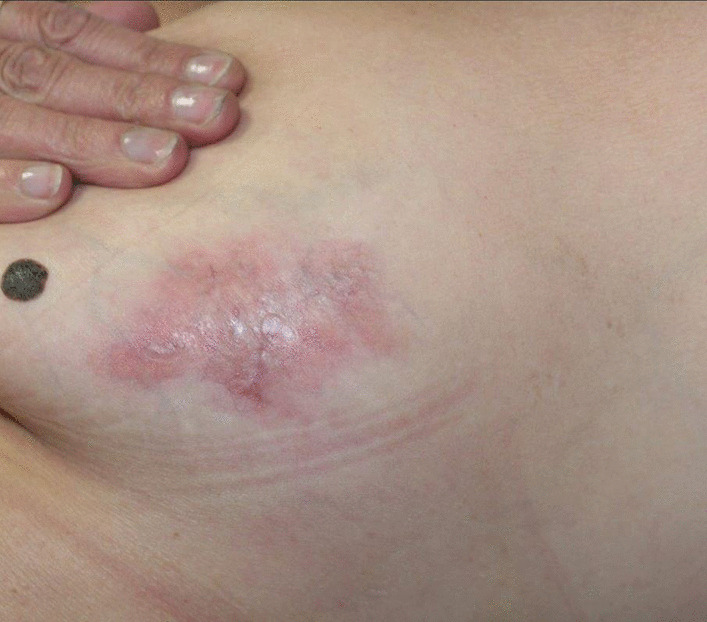
Clinical amelioration of lesions after 6 weeks of treatment

Many skin biopsy specimens were obtained for histopathological, microbiological, and mycological examination. Histology showed palisading non-caseating interstitial epithelioid granulomatous dermatitis with diffuse necrobiosis (Fig. 2). Special stains [Periodic Acid–Schiff stain (PAS), Ziehl, Gram, Grocott] and microbiological and mycological investigations were negative, thus, the infectious origin was ruled out. On the basis of these data, two diagnoses were discussed: NL and specific metastatic CD (MCD), but NL associated with CD was retained, for which we prescribed topical steroids in association with antimalarial drugs with a good response (Fig. 3).

## Discussion

In both NL and specific MCD, non-caseating epithelial-gigantocellular granulomas are observed. Though skin necrobiotic changes are a characteristic feature in NL, they are exceptional in MCD [7]. To the best of our knowledge, there are only three published cases of necrobiotic skin lesions on the lower leg resembling erythema nodosum in MCD [8, 9], which is different from the skin lesions in our patient that are more characteristic of NL of the breast. This location of NL on the breast is extremely rare, as it was reported in only one case a few months following the insertion of a silicone implant (post-mastectomy reconstruction) [10].

## Conclusion

On the basis of this rare original observation, NL without evidence of glucose intolerance should be recognized as a possible cutaneous manifestation or association of CD.

## Data Availability

Please contact the corresponding author for data requests.

## Abbreviations

NL: Necrobiosis lipoidica
CD: Crohn’s disease
MCD: Metastatic Crohn’s disease
PASstain: Periodic Acid Schiff stain
